# Cognitive spare capacity in older adults with hearing loss

**DOI:** 10.3389/fnagi.2014.00096

**Published:** 2014-05-19

**Authors:** Sushmit Mishra, Stefan Stenfelt, Thomas Lunner, Jerker Rönnberg, Mary Rudner

**Affiliations:** ^1^Department of Behavioural Sciences and Learning, Linnaeus Centre HEAD, Swedish Institute for Disability Research, Linköping UniversityLinköping, Sweden; ^2^Department of Clinical and Experimental Medicine, Linköping UniversityLinköping, Sweden; ^3^Eriksholm Research Centre, Oticon A/SSnekkersten, Denmark

**Keywords:** working memory, cognitive spare capacity, updating, inhibition, episodic long-term memory

## Abstract

Individual differences in working memory capacity (WMC) are associated with speech recognition in adverse conditions, reflecting the need to maintain and process speech fragments until lexical access can be achieved. When working memory resources are engaged in unlocking the lexicon, there is less Cognitive Spare Capacity (CSC) available for higher level processing of speech. CSC is essential for interpreting the linguistic content of speech input and preparing an appropriate response, that is, engaging in conversation. Previously, we showed, using a Cognitive Spare Capacity Test (CSCT) that in young adults with normal hearing, CSC was not generally related to WMC and that when CSC decreased in noise it could be restored by visual cues. In the present study, we investigated CSC in 24 older adults with age-related hearing loss, by administering the CSCT and a battery of cognitive tests. We found generally reduced CSC in older adults with hearing loss compared to the younger group in our previous study, probably because they had poorer cognitive skills and deployed them differently. Importantly, CSC was not reduced in the older group when listening conditions were optimal. Visual cues improved CSC more for this group than for the younger group in our previous study. CSC of older adults with hearing loss was not generally related to WMC but it was consistently related to episodic long term memory, suggesting that the efficiency of this processing bottleneck is important for executive processing of speech in this group.

## Introduction

Communication is vital for social participation but may be hampered by adverse listening conditions. Such conditions arise when target speech is accompanied by background noise, possibly including other talkers, and when the listener has a hearing impairment (Mattys et al., 2012). Listening in adverse conditions is associated with individual working memory capacity (WMC, for reviews see Akeroyd, 2008; Besser et al., 2013). Working memory is the ability to maintain and process task-relevant information on line and is necessary for a wide range of complex cognitive activities including speech understanding (Baddeley, 2003). While listening under adverse conditions, individuals may maintain in working memory fragments of spoken information that are not masked by noise and process them to achieve speech understanding (Rönnberg et al., 2013). This processing may involve executive functions including updating to add relevant information to working memory and inhibition to exclude irrelevant information (McCabe et al., 2010; Sörqvist et al., 2010; Rudner et al., 2011; Rudner and Lunner, 2013). There is also evidence that linguistic closure ability supports listening in adverse conditions (Besser et al., 2013; Zekveld et al., 2013) and this may be another process that is recruited into working memory during speech understanding. Because WMC is limited (Baddeley and Hitch, 1974; Just and Carpenter, 1992), it is likely that when cognitive processes are recruited during listening under adverse conditions, fewer resources are available for higher cognitive processing of heard speech (Pichora-Fuller, 2003; Arehart et al., 2013; Mishra et al., 2013a,b; Rudner and Lunner, 2013). In other words, cognitive spare capacity (CSC) is reduced. WMC varies substantially between individuals (Just and Carpenter, 1992) and decreases with age (Nilsson et al., 1997; Nyberg et al., 2012). Thus, it is not surprising that there are individual differences in CSC (Mishra et al., 2013a,b) and there may also be age-related differences. However, work to date has shown that CSC is not reliably associated with WMC (Mishra et al., 2013a,b).

The role of working memory in speech understanding has been conceptualized in the Ease of Language Understanding model (ELU; Rönnberg, 2003; Rönnberg et al., 2008, 2013). The ELU model postulates that in optimal listening situations, when the listener is young and healthy and speech is clear, non-accented and in their native language without background noise or reverberation (Mattys et al., 2012), understanding is implicit or effortless as the incoming signal can be smoothly and rapidly matched with the lexical and phonological representations stored in the mental lexicon in long term memory (LTM, Luce and Pisoni, 1998). In adverse listening conditions, on the other hand, a mismatch may occur between the incoming speech signal and stored representations. This leads to explicit or conscious cognitive processing of the signal in order for language understanding to occur. Such processing may include inhibition, to keep working memory clear of irrelevant information such as speech produced by a non-target talker or other background noise, but also inappropriate inferences about individual words or the gist of the conversation (Pichora-Fuller, 2007). Updating skills are required to correctly prioritize maintenance of information held in working memory in relation to new, incoming information and old information in the form of episodic or semantic representations in LTM. Linguistic closure skills are required to make efficient inferences concerning the lexical and semantic identity of spoken information on the basis of the ongoing processing of fragments of speech information held in working memory (Besser et al., 2013).

Aging is associated with both sensory and cognitive decline. Older adults usually have raised hearing thresholds and reduced spectral and temporal resolution (Gordon-Salant, 2005). In addition, they may have temporal auditory processing deficits (Pichora-Fuller and Souza, 2003). Further, reduced cognitive resources may make speech recognition more difficult for older adults compared to young (Pichora-Fuller and Singh, 2006; Mattys et al., 2012), due to impoverished encoding of the target stimuli into episodic memory, despite adequate recognition (Pichora-Fuller et al., 1995; Heinrich and Schneider, 2011; Sörqvist and Rönnberg, 2012). When target stimuli are presented against a speech-like background, older individuals encounter additional problems because they are not as efficient at processing target information present in the gaps in the speech-like masker (George et al., 2007), nor at suppressing automatic linguistic processing of the irrelevant speech (Ben-David et al., 2012). Thus, factors relating to both sensory and cognitive decline are likely to increase the risk of mismatch between speech input and representations in LTM, leading to a relative decrease in CSC for older adults with hearing loss compared to younger adults with normal hearing under similar environmental circumstances. Further, there are differences in the way younger and older adults deploy their cognitive resources during speech understanding (Pichora-Fuller et al., 1995; Murphy et al., 2000; Wong et al., 2009) and these are likely to be compounded by working memory load or the amount of information that needs to be held in working memory at any one time (Reuter-Lorenz and Cappell, 2008). In particular, older adults may make full use of all available cognitive resources when load is still relatively low, while younger adults still have spare capacity to cope with higher load.

Recent work has demonstrated that a greater degree of hearing loss in older adults is associated with poorer LTM (Lin et al., 2011; Rönnberg et al., 2011). It has been suggested that the mechanism behind this may be related to the mismatch function as described by the ELU model (Rönnberg et al., 2013). In particular, if mismatch occurs regularly over an extended period of time, LTM may be accessed less frequently leading to disuse and less efficient LTM function (Rönnberg et al., 2011; Classon et al., 2013). As we have seen, mismatch frequency in any given situation may be related to both sensory and cognitive factors and in older adults, general cognitive slowing may make it harder to resolve mismatch (Pichora-Fuller, 2003). Thus, relatively well-preserved cognitive processing speed and LTM efficiency are likely to enhance CSC in older adults with hearing loss.

While noise, hearing impairment and increasing age make it harder to understand speech, viewing the speaker's face may enhance the listener's ability to segregate the speech signal from the noise, thereby making it easier to understand (Campbell, 2009). It has been suggested that the presence of visual cues helps the listener, especially older adults, to attend to the incoming signal at the most critical time for encoding (Helfer and Freyman, 2005). This results in less signal uncertainty and fewer cognitive demands in anticipating target stimuli than in the absence of visual cues (Besle et al., 2004; Moradi et al., 2013). Thus, visual cues seem to reduce the cognitive demands of listening in noise, especially for older adults. Seeing the talker's face during encoding improves WMC and in older adults has been shown to reduce neural activation, indicating a processing benefit (Frtusova et al., 2013).

To investigate CSC and assess the effect of noise, memory load and visual cues on executive processing of speech, we developed a test of CSC, CSCT. In the CSCT, participants listen to lists of spoken two-digit numbers presented serially with (AV) and without (A-only) a video of the talker's face. Presentation may take place in quiet or in background noise adjusted to a level at which good stimulus intelligibility is maintained. At the end of each list, participants recall two (low memory load) or three numbers (high memory load) depending on instructions eliciting one of two different executive functions (updating and inhibition). In young adults with normal hearing thresholds, CSCT performance is generally better for inhibition than updating, and low than high memory load (Mishra et al., 2013a,b). Steady-state background noise, which may be described as a rushing sound, reduces CSCT scores even when intelligibility is high (Mishra et al., 2013b). However, at the same SNR, speech-like background noise that consists of unintelligible speech fragments pieced together, does not seem to reduce CSCT scores in young adults with normal hearing thresholds, possibly because executive skills allowing dynamic tracking of target speech and simultaneous suppression of non-target speech leading to richer memory representation (Mishra et al., 2013b; Zion Golumbic et al., 2013). Although visual cues restore performance in steady-state background noise, probably by facilitating segregation of target speech from noise (Mishra et al., 2013b) they reduce performance in quiet, probably by causing distraction when the auditory signal provides adequate information to solve the CSC task (Mishra et al., 2013a,b). CSCT performance in young adults with normal hearing is predicted by updating skills but not, generally speaking, by WMC (Mishra et al., 2013a,b).

In the present study, we investigated CSC in adults with hearing loss who were older than the participants in our previous CSC studies (Mishra et al., 2013a,b). The CSCT and a cognitive test battery were administered. In the CSCT, audibility was restored by individualized amplification. We expected to find an overall pattern of results generally similar to that in our previous CSC studies but with stronger effects of load and noise manipulations due to an overall decrease in CSC attributable to age and hearing loss. Specifically, we expected better performance in inhibition than updating conditions and in low than high memory load conditions, as well as better performance in quiet than in noise. However, we also expected speech-like as well as steady-state noise to reduce CSCT scores due to age-related reduction in executive skills (Nyberg et al., 2012) and hearing-loss-related reduction in the ability to segregate target from background speech-like noise (Festen and Plomp, 1990; George et al., 2006, 2007; Lorenzi et al., 2006; Ben-David et al., 2012). We expected visual cues to restore performance in noise conditions (Frtusova et al., 2013). Because younger and older adults use different strategies to deploy cognitive resources, we expected to find a different pattern of associations between CSCT performance and the cognitive test battery (Pichora-Fuller et al., 1995; Murphy et al., 2000; Reuter-Lorenz and Cappell, 2008; Wong et al., 2009; Avivi-Reich et al., 2014). In particular we expected better CSC to be associated with faster cognitive processing speed (Pichora-Fuller, 2003) and better LTM (Rönnberg et al., 2011).

## Methods

### Participants

**Figure 1:**
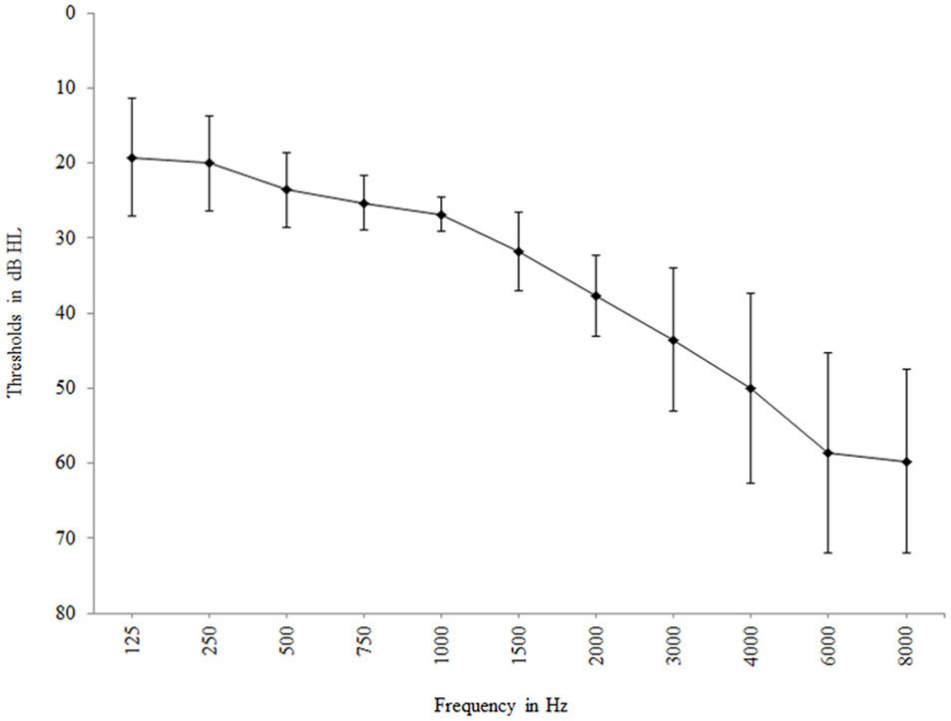
**Mean hearing threshold at the 11 measured frequencies**. Error bars represent standard deviation.

Twenty-seven adults with mild-to-moderate hearing loss and no reported tinnitus consented to participate in the study. They were all recruited from the hearing clinic at Linköping University Hospital, Sweden. Two participants opted to drop out of the testing and one participant was excluded due to poor vision. Thus, 24 participants (61–75 years of age, *M* = 69, *SD* = 4.7), 14 males and 10 females, completed the testing. All participants had sensorineural hearing loss (Air-Bone gap < 10 dB HL) and the average pure-tone threshold (PTA_4_) across 0.5, 1, 2, and 4 kHz was 34.5 dB HL (*SD* = 3.6), see Figure 1. An epidemiological study covering the same area showed that 73.1% of the population in the age range of 70–80 years and 42.1% in the age range of 60–70 years had at least a mild hearing loss (Johansson and Arlinger, 2003). In the present study, all participants had mild (PTA_4_: 26–40 dB HL; WHO, 2013) hearing loss, except for one participant aged 74 years who had moderate (PTA_4_: 41–60 dB HL; WHO, 2013) hearing loss. Hearing thresholds of all participants at all four frequencies were within one standard deviation of population means for the age group reported by Cruickshanks et al. (1998). Thus, hearing status was representative for their age group. The participants reported that their hearing loss was acquired post-lingually and that they did not have any otological, psychological or neurological problems. Three participants reported that they used hearing aids occasionally while the others were non-users. Visual acuity after correction was normal as measured using the Jaeger eye chart (Weatherly, 2002). Ethical approval for the study was obtained from the regional ethical review board.

### Cognitive spare capacity test (CSCT)

The CSCT is an auditory working memory task that systematically manipulates storage and executive processing demands along with modality of presentation and noise conditions (Mishra et al., 2013a,b).

#### Material

The CSCT stimuli consisted of AV and A-only recordings of Swedish two digit numbers 13–99 (Mishra et al., 2013a,b). The numbers were spoken by two native Swedish speakers, one male and one female, with no distinctive dialect. The levels of the numbers were equated for equal intelligibility in steady-state noise (Mishra et al., 2013b). The two-digit numbers were arranged serially in 48 lists of 13 numbers each. Numbers were never repeated within lists or condition and numbers spoken by same speaker were repeated between two and eight times across all the lists. Half of the lists included AV stimuli and the other half included A-only stimuli. Within each modality, 12 lists were used for each of the two CSCT tasks. The serial position of the target numbers within the lists and contingent task demands were balanced across the lists. For more details of CSCT materials, see Mishra et al. (2013a).

#### Noise

Stationary and modulated noises were used. The stationary noise was a steady-state speech-weighted (SSSW) noise, having the same long term average spectrum as the stimuli (numbers). The modulated noise was the International Speech Testing Signal (ISTS), which contains concatenated short segments of speech in six different languages (Holube et al., 2010) and is thus speech-like but unintelligible.

#### Individualizing SNR and amplification

Audibility is a key factor for speech understanding (Humes, 2007) and thus for optimizing the task in CSCT. Therefore, the CSCT lists were presented with amplification compensating for the hearing loss of the participants and at individualized SNR ensuring an intelligibility level of around 90% for the SSSW noise. An adaptive procedure implemented in MATLAB (Version 2009b) was used to determine the individualized SNR for presentation of the CSCT. This procedure was implemented in two steps and was based on the stimulus materials (numbers) in the A-only modality and the SSSW noise (Mishra et al., 2013b). In the first step, a number was presented at an SNR of 5 dB and the participant was instructed to repeat the number he or she heard. Then the noise was increased by steps of 3 dB each time the number was repeated correctly. When the participant made an incorrect response, the SNR was improved by 1 dB and a new number was presented. Thirty such randomly selected numbers, were presented consecutively with a step-size of 1 dB for the level of noise to determine the 84% intelligibility level in a four up-one down procedure (Levitt, 1971). In the second step, in order to achieve an intelligibility level of approximately 90% in SSSW noise, the SNR obtained for 84% intelligibility was increased by 0.5 dB for each individual. To verify the individual intelligibility at this new SNR in both SSSW and ISTS noises, 60 randomly selected numbers were presented at the set SNR in SSSW and another 60 numbers in ISTS noise to measure the intelligibility levels for both noise types. In the CSCT, the ISTS noise was presented at exactly the same SNR as the SSSW noise.

The signal (numbers and noise) for all speech in noise tests with auditory presentation was amplified using the Cambridge prescriptive formula (Cameq) for linear hearing aids (Moore and Glasberg, 1998). This amplification was implemented in a master hearing aid (MHA) system (Grimm et al., 2006). The participant's audiogram was used to set the gain according to the Cameq fitting rule giving individual amplification for each participant.

#### Tasks

There are two different CSCT tasks, updating and inhibition which are designed to engage the corresponding executive functions. In the updating task, the participants are asked to recall either the highest or the lowest value item spoken by the male and female speaker in the particular list. Thus, each time an item is presented that meets the criterion, the participant has to encode this item into working memory and discard any previous item which it replaces. In the inhibition task, the participants are asked to recall either two odd or even value items spoken by a particular speaker. Thus, the participant has to inhibit encoding of items produced by a particular talker while monitoring items of the desired parity. These tasks are performed with either AV or A-only stimulus presentation. After each list, the participant is requested to report two specified list items, depending on the task to be performed. In half of the trials, which were the low memory load trials, only these two numbers are reported. The two specified numbers never include the first item in the list. In the other half of the trials, the participant is requested to report the first number in the list along with the two specified items, i.e., three numbers in total need to be held in working memory but only two of them are subject to executive processing. These are the high memory load trials. For these trials, the first number (dummy item) is not included in the scoring. Thus, all scoring in the CSCT is based on correct report, in any order, of two numbers.

#### Experimental design

All participants performed the CSCT with stimulus presentation in quiet (no noise), SSSW noise and ISTS noise. Thus, there were a total of 24 conditions of presentation in the CSCT with two executive tasks (Updating, inhibition), two memory loads (High, low), two modalities (AV, A-only) of presentation and three noise conditions (quiet, SSSW and ISTS noise) in a 2 × 2 × 2 × 3 design.

#### Administration of CSCT

The CSCT was administered using DMDX software (Forster and Forster, 2003; Mishra et al., 2013a,b). The participants performed the CSCT under 12 different conditions per executive task in separate blocks and hence two lists per condition were tested. The order of the conditions was pseudo-randomized within the two task blocks and balanced across the participants. For the noisy conditions, the noise sound files were played together with the AV and A-only stimulus files in DMDX. The noise onset was 1 s prior to onset of stimulus and the noise offset was at least 1 s after the stimulus offset. The lists of numbers were always presented at 65 dB SPL and the level of the noise was varied depending upon the individualized SNR level before individualized amplification for hearing loss. The same individualized SNR was used for all noisy trials. Across all the conditions (noisy or quiet), the duration of presentation of each number list was 33 s in AV and A-only modality. The visual stimuli were presented using a computer with screen size of 14.1 inches and the amplified auditory stimuli were presented through Sennheiser HDA 200 headphones.

The participants were provided with written instructions for the particular executive task before each of the blocks and the instructions were also elaborated orally. In addition to this, before each list the participant was prompted on the computer screen as to which version of the executive task was to be performed, what the modality was and whether to remember two or three numbers (high or low load). The task prompt remained on screen until the participant pressed a button to continue to the test. At the end of each list, an instruction “Respond now” appeared on the screen and the participant was required to say the target numbers. Corrections to reported numbers were allowed and responses were audio recorded. The participant then pressed another button when they were ready to continue. All the participants practiced each task with two lists before doing the test. The participants were specifically instructed to keep looking at the screen during stimulus presentation. This applied even during presentation in the A-only modality where a fixation cross was provided at center screen. If they looked away from the screen, the test was stopped after presentation of the list and the participants were reinstructed to keep looking at the screen.

### Cognitive test battery

#### Reading span

The participants read series of sentences which appeared on the computer screen one at a time (Daneman and Carpenter, 1980; Rönnberg et al., 1989). Each series consisted of three to six sentences presented in increasing series length while each sentence consisted of three words. There was an interval of 50 ms between words and each word was shown for 800 ms. Half of the sentences were coherent and half were absurd. After each sentence, the participant was given 1.75 s to judge the semantic coherence of the sentence before the next once appeared. The participant responded “yes” (if the sentence was coherent) or “no” (if the sentence was absurd). At the end of each series of sentences, the participants were prompted by an instruction on the screen to recall either the first or the last word of all the sentences in the series in the order in which they appeared on the screen. All participants practiced with a series of three sentences before the actual testing and the practice was repeated if necessary. There were a total of 54 sentences in the actual test. The dependent measure was the total number of words correctly recalled in any order.

#### Text reception threshold (TRT)

The TRT (Zekveld et al., 2007) provides a measure of linguistic closure (Besser et al., 2013). In TRT, sentences in red appear word by word on a computer screen partially masked by black bars. The participants are asked to guess the sentence correctly. A Swedish version of the TRT, using the Hearing in Noise Test (HINT) sentences (Hällgren et al., 2006) was used in the present study. First, a practice list with 20 sentences was presented which was followed by the actual testing where two similar lists were presented. All words remained on the screen until the sentence was completed and after presentation of the last word the sentence remained visible for 3.5 s. The presentation rate of the words in each sentence was equal to the speaking rate in a corresponding speaker file. If the participants were unsuccessful in reading the sentence, feedback was provided. A one-up-one-down adaptive procedure with a step-size of 6% was applied to target percentage of unmasked text required to read 50% of the sentences entirely correctly. The average percentage of unmasked text from the two lists of sentences was used as dependent variable.

#### Letter memory

The letter memory task (Morris and Jones, 1990; Miyake et al., 2000) was presented using a DMDX platform. Series of consonants were presented at the center of the computer screen. The participants were asked to hold the four most recent letters in mind and then prompted to say them at the end of each series. Responses were audio-recorded. In order to ensure that the participant followed the instructed strategy and continuously updated working memory until the end of the trial, series length was randomized across trials. Two series consisting of seven and nine letters were presented as practice and the actual testing consisted of 12 series varying in length between five and 11 items. The practice sequences were repeated until participants followed the instructed strategy. The number of consonants correctly recalled irrespective of order was the independent measure of updating.

#### Simon

A visual analog of the Simon task (Simon, 1969; adapted from Pratte et al., 2010), consisting of presentation of red and blue rectangular blocks on a computer screen, was used to provide a measure of the inhibition. The blocks appeared on the left or the right of the computer screen successively at intervals of 2 s. The participants were instructed to respond as quickly as possible while maintaining accuracy by pressing a button on the right hand side of the screen when they saw a red block and when they saw a blue block they pressed a button on the left hand side of the screen. A total of 16 blocks were presented using DMDX. No practice item was provided. When the spatial position of the stimulus and correct response key coincided, the trial was termed congruent otherwise incongruent. The participant had to ignore the spatial position in which the block appeared in the task. The difference in reaction time between the incongruent and congruent trials was taken as the dependent variable. The mean reaction time obtained on the congruent trial of the Simon task for each participant was taken as a measure of processing speed.

#### Delayed recall of reading span

A delayed free recall of the reading span test (Mishra et al., 2013b) was used to measure the episodic LTM of the participants. In this test the participants were asked to recall words or sentences remembered from the reading span test after approximately 60 min, without forewarning. During the 60 min, the participants performed the other tests in the cognitive test battery. The score in the delayed free recall of the reading span test was the total number of words recalled by the participant, irrespective of the order and the performance in the reading span test. The participants did not have any time restriction to recall the words or sentences.

### Procedure

The testing was conducted in two sessions. All auditory testing took place in a sound-treated booth with the participants facing the computer screen. Each session took approximately 90 min. The participants, on arriving for the testing, were fully briefed about the study and a consent form was signed. The participants were provided with written instructions about the test and instructions were verbally elaborated if needed. All the participants underwent vision screening and audiometric testing in the audiometric booth. In a separate room, the reading span test was administered followed by the Simon task, the letter memory test and the TRT test. Individual SNRs for the CSCT were determined and the delayed recall of the reading span test was the last test of the first session. In the second session, CSCT was conducted. The participants were allowed to take breaks between the tests.

### Data analysis

To ensure that the performance of the three occasional hearing-aid users did not differ from that of the other participants, it was checked that their scores in the various tests were within one standard deviation of the mean score of the participants who did not use hearing aids. An overall repeated measures analysis of variance (ANOVA) on the CSCT scores was conducted. The inter-correlation among the cognitive tests and the association between cognitive functions and CSCT was assessed using Pearson's correlations.

## Results

### Intelligibility

The mean SNR for CSCT presentation in noise was −0.17 dB (*SD* = 1.39). The mean intelligibility levels were 94.5% (*SD* = 3.0) and 88.3% (*SD* = 3.0) for the SSSW and ISTS noise, respectively. The difference between these levels was statistically significant, *t*_(46)_ = 7.05, *p* < 0.01.

### Cognitive spare capacity test (CSCT)

**Figure 2:**
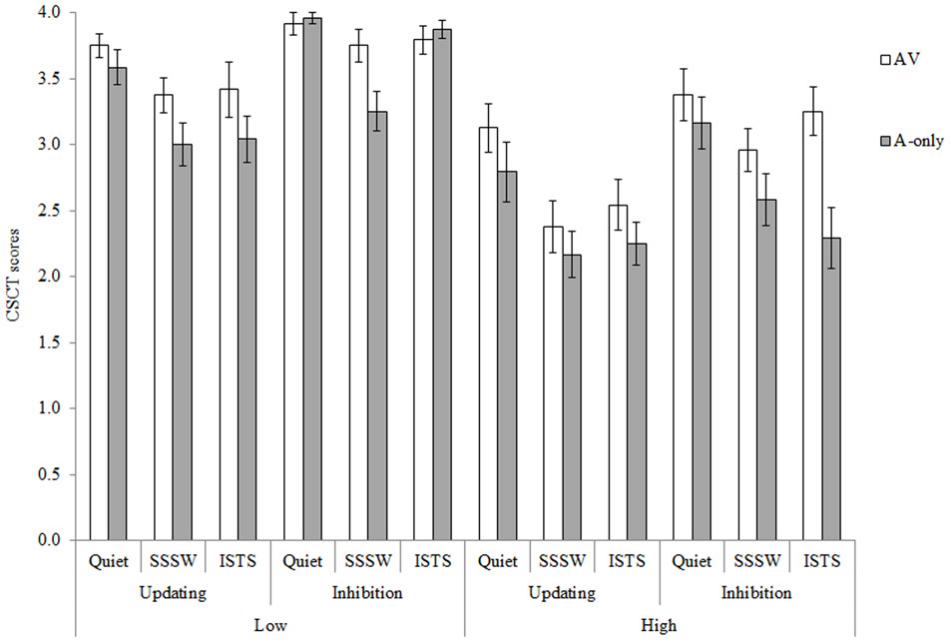
**Mean CSCT raw scores for the AV (unfilled bars) and A-only (filled bars) modalities of presentation in the high and low memory load conditions of the updating and inhibition tasks in the three noise conditions**. Error bars represent standard error.

Mean raw scores are shown in Figure 2. The maximum possible score per condition was four, as two lists were presented per condition. Performance in the inhibition task in the low memory load for quiet and ISTS noise conditions approached ceiling. Hence, all analyses of CSCT data were conducted on the rationalized arcsine-transformed scores (Studebaker, 1985) to counteract data skewing. Performance on the updating and inhibition subsets correlated significantly, (*r* = 0.57, *p* < 0.01), confirming internal and construct validity.

The overall repeated measures ANOVA revealed main effects of all four factors: executive function, *F*_(1, 23)_ = 30.00, *MSE* = 0.23, *p* < 0.001, showing higher CSCT scores in inhibition than updating conditions; memory load, *F*_(1, 23)_ = 71.71, *MSE* = 0.35, *p* < 0.001, showing higher CSCT scores in low than high memory load conditions; modality, *F*_(1, 23)_ = 26.23, *MSE* = 0.14, *p* < 0.001, showing CSCT scores were higher in AV than A-only conditions and noise, *F*_(2, 46)_ = 23.78, *MSE* = 0.18, *p* < 0.001. Pair-wise comparisons with Bonferroni adjustment for multiple comparisons were conducted in order to identify statistically significant differences in performance between the three noise types. They showed that CSCT performance in quiet was better than in both ISTS and SSSW noise (*p* < 0.05), but there was no difference between performance in ISTS and SSSW noise (*p* = 0.13). All main effects were in line with our prediction. It should be borne in mind here that intelligibility was significantly higher in SSSW noise than in ISTS noise. To test whether this difference in intelligibility influenced memory performance, we examined whether there was a difference in recall of the first list item in the high memory load conditions when items were presented in SSSW noise compared to ISTS noise. There was no statistically significant difference, *t*_(46)_ = 0.01, *p* > 0.05. This suggests that the lack of difference in CSCT performance in SSSW and ISTS noise is not an artifact of intelligibility differences. There were no statistically significant Two-Way or Three-Way interactions.

### Cognitive test battery

Table 1 shows the mean performance and standard deviation in the cognitive test battery. In the reading span semantic judgment task, the mean score was 50.5 (*SD* = 3.20) out of 54 possible responses, demonstrating adherence to instructions. We excluded the delayed recall of reading span score of one participant that was more than two standard deviations above the mean score.

**Table 1:** **Mean performance and standard deviation (*SD*) in the cognitive test battery and results of two-tailed independent sample *t*-tests with young adults with normal hearing thresholds included in the reanalysis**.

**Cognitive test**	**Units**	**Mean**	***SD***	***t*, *p***
Reading span	Words recalled (max 54)	21.38	4.61	4.8, 0.00
Letter memory	Letters recalled (max 48)	37.79	4.09	3.65, 0.00
Simon	Difference in reaction time in ms between incongruent and congruent trials	133.46	82.84	2.25, 0.03
Simon congruent trials	Reaction time in ms	628.29	140.12	2.11, 0.04
TRT	Percentage unmasked text	52.53	4.62	3.23, 0.00
Delayed recall of reading span	Words recalled	5.90, *n* = 23	2.90	4.91, 0.00

Table 2 shows the correlations among the cognitive tests, PTA_4_ threshold and age. PTA_4_ threshold was associated with age, and reading span was associated with letter memory and delayed recall of reading span, see Table 2.

**Table 2:** **Coefficients of correlations (Pearson's r) between age, average pure tone thresholds across the four frequencies 0.5, 1, 2, and 4 kHz (PTA_4_) and cognitive test scores**.

	**PTA_4_**	**Reading span**	**Letter memory**	**Simon**	**TRT**	**Delayed recall of reading span**	**Simon congruent trials**
Age	0.51^*^	−0.12	−0.11	−0.28	0.34	−0.19	0.35
PTA_4_		−0.11	−0.18	−0.29	0.23	−0.27	−0.03
Reading span			0.51^*^	−0.27	0.02	0.44^*^	−0.03
Letter memory				0.21	−0.36	0.36	−0.12
Simon					−0.40	−0.23	−0.05
TRT						−0.07	0.37
Delayed recall of reading span							−0.19

* *Correlation is significant at the 0.05 level (two-tailed)*.

Table 3 shows the overall and factor-wise association between CSCT performance and cognitive skills. Delayed recall of reading span was associated with CSCT irrespective of how scores were split. Letter memory was associated with the overall CSCT score as well as performance in CSCT updating, A-only, high memory load, quiet and ISTS noise conditions. There was no statistically significant correlation between Simon and the inhibition conditions of the CSCT (*p* = 0.63). However, TRT was associated with performance in inhibition conditions. A higher score in TRT indicates poorer performance. Therefore, the negative correlation shows that better TRT performance is associated with better CSCT performance in the inhibition condition. Working memory as measured by the reading span test correlated significantly with the performance of CSCT in ISTS noise conditions. Reaction times on the congruent trials of the Simon task, which was our measure of processing speed, did not correlate significantly with CSCT performance. We did not include a measure of motor processing speed in the present study and thus we cannot exclude the possibility that cognitive processing speed was confounded by differences in motor skills.

**Table 3:** **Coefficients of correlations (Pearson's r) between factorwise CSCT scores and cognitive test scores**.

**CSCT**	**Reading span**	**Letter memory**	**Simon**	**TRT**	**Delayed recall of reading span**	**Simon congruent trials**
Overall	0.29	0.51^*^	−0.05	−0.36	0.64^**^	−0.29
Updating	0.33	0.50^*^	−0.16	−0.23	0.69^**^	−0.18
Inhibition	0.18	0.40	0.11	−0.42^*^	0.41^*^	−0.33
AV	0.29	0.38	0.04	−0.36	0.61^**^	−0.33
A−only	0.25	0.57^**^	−0.12	−0.31	0.60^**^	−0.22
Low load	0.21	0.26	−0.29	−0.13	0.42^*^	−0.35
High load	0.17	0.50^*^	0.03	−0.39	0.58^**^	−0.22
Quiet	0.27	0.46^*^	−0.03	−0.29	0.77^**^	−0.26
SSSW	−0.09	0.18	−0.07	−0.24	0.66^**^	−0.24
ISTS	0.44^*^	0.54^**^	−0.15	−0.32	0.77^**^	−0.25

* *Correlation is significant at the 0.05 level (two-tailed)*.

** *Correlation is significant at the 0.01 level (two-tailed)*.

## Discussion

In the present study we investigated CSC for speech heard in quiet and in noise in adults with hearing loss in AV and A-only modality of presentation. We did this by administering the CSCT (Mishra et al., 2013a,b), a test of CSC that measures individuals' ability to perform executive processing of heard material at different memory loads. The CSCT was presented with individualized amplification in SSSW and ISTS noise as well as in quiet. In the two noise conditions, it was presented at an estimated speech intelligibility level of approximately 90%.

### CSCT performance

In line with predictions, performance was better when memory load was low compared to high and when the task involved inhibitory processing rather than updating. Also in line with expectation, performance was better in quiet than in noise and in the AV than A-only modality in noise and quiet. This was contrary to our previous study (Mishra et al., 2013a,b) in which visual cues hindered performance in the quiet conditions. In Mishra et al. (2013b) when young adults performed CSCT in quiet and in noise, the Two-Way interaction between modality and noise was significant, revealing higher CSCT scores in the A-only compared to the AV modality in quiet and the opposite in noise. Although the finding of poorer performance with visual cues is unexpected in relation to much of the perceptual and cognitive literature, it is in line with the results of recent studies showing that superfluous information carried in the visual stream may reduce performance on a dual task paradigm (Fraser et al., 2010; Gosselin and Gagné, 2011). This phenomenon may arise when executive demands make it difficult to prioritize task-related processing in the presence of low priority stimuli (Lavie, 2005). We proposed that in conditions where all the information needed to solve the task was available in the auditory signal, assuming optimum speech intelligibility for participants with normal hearing listening in quiet, the visual cues constituted a distraction (Mishra et al., 2013a,b). It has been shown that the presence of visual cues reduces the cognitive demands for perception of speech (Besle et al., 2004; Moradi et al., 2013). The reduction in cognitive demands leads to better representation of the target signal in memory (Pichora-Fuller et al., 1995; Heinrich and Schneider, 2011). Even in quiet, the signal is degraded for older adults with hearing loss due to receiver limitation (Mattys et al., 2012). Hence, we suggest that even in quiet conditions, seeing the talker's face helps older individuals with hearing loss to form better cognitive representations of spoken words leading to higher performance in AV modality, possibly by viseme and phoneme information working together (Feld and Sommers, 2009).

CSCT performance was poorer in both types of noise than in quiet, whereas in our previous study, only SSSW reduced CSCT performance for young adults (Mishra et al., 2013b). It has been demonstrated that being older and having a hearing loss are associated with poorer speech segregation, especially when noise is speech-like (Festen and Plomp, 1990; George et al., 2006, 2007; Ben-David et al., 2012), probably because relevant cognitive functions are less efficient and deployed differently (Pichora-Fuller et al., 1995; Murphy et al., 2000; Reuter-Lorenz and Cappell, 2008; Wong et al., 2009). We suggest that this is also the cause of lower CSC in older adults with hearing loss.

### Executive processing in CSCT

To check that the CSCT tasks tapped into the intended executive functions, we investigated correlations with the cognitive test battery. The performance on the updating task of CSCT collapsed across other factors correlated with performance on the letter memory task, confirming previous results (Mishra et al., 2013a,b) and showing that the updating task of the CSCT does tap updating skills. However, as we found in our previous study using the same paradigm (Mishra et al., 2013b) the correlation between performance on the inhibition task of the CSCT and the Simon task was not statistically significant. In both studies, noise was introduced for two out of three lists in an unpredictable manner. Thus, inhibition skills available for solving the executive task may have been reduced, even for the lists presented in quiet. An alternative explanation could be that the Simon task does not measure inhibition. However, we find this explanation implausible as we found a significant correlation (*r* = −0.46) between Simon and CSCT performance under inhibition conditions in a study in which noise was not presented in any condition (Mishra et al., 2013a). Depletion of inhibition skills was probably compounded in the present study by the reduced executive skills of the participants (c.f. Ben-David et al., 2012). The statistically significant association between CSCT performance in inhibition conditions and TRT performance, which provides a measure of linguistic closure, found in the present study for adults with hearing loss as well as in our previous study for adults without hearing loss (Mishra et al., 2013b), suggests that linguistic closure may compensate for depleted executive skills. Zekveld et al. (2012) pointed out that TRT predicted speech perception in noise when the irrelevant cues for speech understanding had to be disregarded or inhibited. Similarly in the inhibition task of CSCT, the numbers of same parity spoken by the opposite gender had to be disregarded. Further studies should investigate the interplay of executive function and linguistic closure ability during higher processing of speech.

The main effects of executive function, memory load and modality revealed that CSCT performance was lower when the task was updating, memory load was high and the visual cues were absent. Under these conditions, CSCT performance consistently correlated with updating skills. Thus, good updating skills seem to be particularly important for higher level processing of speech when task demands are particularly high for older individuals with hearing loss. This finding contrasts with the finding of our previous study using the same paradigm (Mishra et al., 2013b) in which good updating skills were associated with good CSCT performance in almost all conditions. In the present study, consistent associations between CSCT performance and delayed recall of reading span were found across the board.

### Role of working memory and episodic long-term memory (LTM) in CSCT performance

Performance in CSCT was not significantly associated with performance in the reading span test except in the ISTS noise conditions. Previous work has shown that speech recognition in modulated noise, especially speech noise, is associated with WMC (Zekveld et al., 2013). This may be because listening in modulated noise involves integrating fragments of information available in the dips in the noise (Lunner, 2003). Hence, it is not surprising to find that CSCT performance in ISTS noise but not in SSSW noise was associated with WMC as measured by reading span performance. In line with our prediction, better episodic LTM, as measured by delayed recall of reading span, was consistently associated with better CSCT performance. Recent work has demonstrated that older adults with hearing loss may have a limited LTM (Lin et al., 2011; Rönnberg et al., 2011). Thus, LTM may form a processing bottleneck for this group and individuals with more efficient LTM are likely be able to process speech with fewer demands on cognitive resources (Rönnberg et al., 2013), resulting in larger CSC. This interpretation is also in line with notion that there are age-related changes in depth of processing of heard material (Craik and Rose, 2012). In older adults, a general cognitive slowing makes matching of the incoming signal with representations stored in LTM more effortful and susceptible to errors (Pichora-Fuller, 2003) and hence faster processing speed may lead to higher scores in CSCT. However, in the present study we did not find any such evidence.

To compare the performance of the participants with hearing impairment in the present study with that of participants without hearing impairment in a previous study (Mishra et al., 2013b), a reanalysis was performed. We expected that the CSCT and cognitive test scores of the participants in the present study would be lower than those of younger adults with normal hearing in our previous study. Further, we expected the CSCT scores of the participants in the present study would be more influenced by high memory load, noise (Pichora-Fuller et al., 1995; Heinrich and Schneider, 2011) and the absence of visual cues (Frtusova et al., 2013).

## Reanalysis

In the present study, the participants were older adults with hearing loss. The hearing thresholds of these participants were similar to those of similar age cohorts in epidemiological studies (e.g., Cruickshanks et al., 1998; Johansson and Arlinger, 2003), suggesting that the hearing loss was age-related. Older adults with normal hearing were not selected in this study because such a group is not representative of the population of older adults. Thus, the reanalysis explores the effect of aging and concomitant auditory decline on CSCT performance. This is achieved by comparing the data of the present study with those of a previous study (Mishra et al., 2013b) where CSCT was administered to young adults with normal hearing.

### Methods

#### Participants

The reanalysis included the participants in the present study and the 20 young adult participants in a previous study (Mishra et al., 2013b). The young adults were 19–35 years of age (*M* = 25.9; *SD* = 4.4) and the mean PTA_4_ 3.2 dB HL (*SD* = 3.2). There were statistically significant differences in age (*t* = 30.77, *p* < 0.01) and PTA_4_ (*t* = 23.25, *p* < 0.01) with the participants in the present study being older and having higher hearing thresholds.

#### Material and noise

The material consisted of lists of two digits numbers presented in AV and A-only modality which were prepared into lists, each containing 13 numbers. The lists were presented in quiet and in SSSW and ISTS noise at intelligibility levels approximating 90%. The material and noise were identical in both the studies.

#### Individualizing SNR and amplification

The methods used for individualizing SNR were identical for the two groups. However, because the younger participants did not have any hearing loss they were not provided with amplification.

#### Tasks, experimental design, and procedure

In both the studies, the testing was conducted in two sessions. In the first session the audiometric testing, vision screening, cognitive test battery and individualized SNR for CSCT presentation was determined. The cognitive test battery consisted of reading span, Simon, letter memory, TRT, and delayed recall of reading span. The order of testing was also identical in both studies. For CSCT, in both studies, order of the conditions was pseudo-randomized within the two executive task blocks and was balanced across the participants in the same manner. Hence, the task, experimental design and procedure were identical in both studies.

#### Data analysis

The data were analyzed using a mixed repeated measures ANOVA on CSCT scores with the two groups of participants as a between subjects variable. Where significant interactions were obtained in ANOVAs, the simple main effects observed were investigated using *post-hoc* Tukey's Honestly Significant Difference (HSD) test. In order to test simple main effects in accordance with our a-priori hypothesis, planned comparisons were carried out. Independent sample *t*-tests were used to compare performance across groups on the cognitive test battery.

### Results

#### Intelligibility

The mean SNR for CSCT presentation in noise for the older adults with hearing loss was −0.17 dB (*SD* = 1.39) and for the young adults, the mean SNR of presentation was −2.17 dB (*SD* = 0.85; Mishra et al., 2013b). The mean intelligibility levels for older adults with hearing loss were 94.5% (*SD* = 3.0) and 88.3% (*SD* = 3.0) for the SSSW and ISTS noise respectively. For the young adults, the mean intelligibility level was 93.8% (*SD* = 3.0) and 92.3% (*SD* = 2.9) for the SSSW and ISTS noise, respectively, (Mishra et al., 2013b). A mixed repeated measures ANOVA conducted on the actual intelligibility levels in SSSW and ISTS noise showed that there was a main effect of noise, *F*_(1, 42)_ = 43.86, *MSE* = 0.01, *p* < 0.001, indicating that the intelligibility levels were higher in SSSW compared to ISTS noise. The Two-Way interaction between group and noise was also statistically significant, *F*_(1, 42)_ = 12.49, *MSE* = 0.01, *p* = 0.001. *Post-hoc* Tukey HSD tests assessing this interaction revealed that in the SSSW noise there was no statistically significant difference in intelligibility levels between groups, but in ISTS noise, the intelligibility level for the participants in the present study was significantly lower than that for the young adults with normal hearing, statistically.

#### CSCT

**Figure 3:**
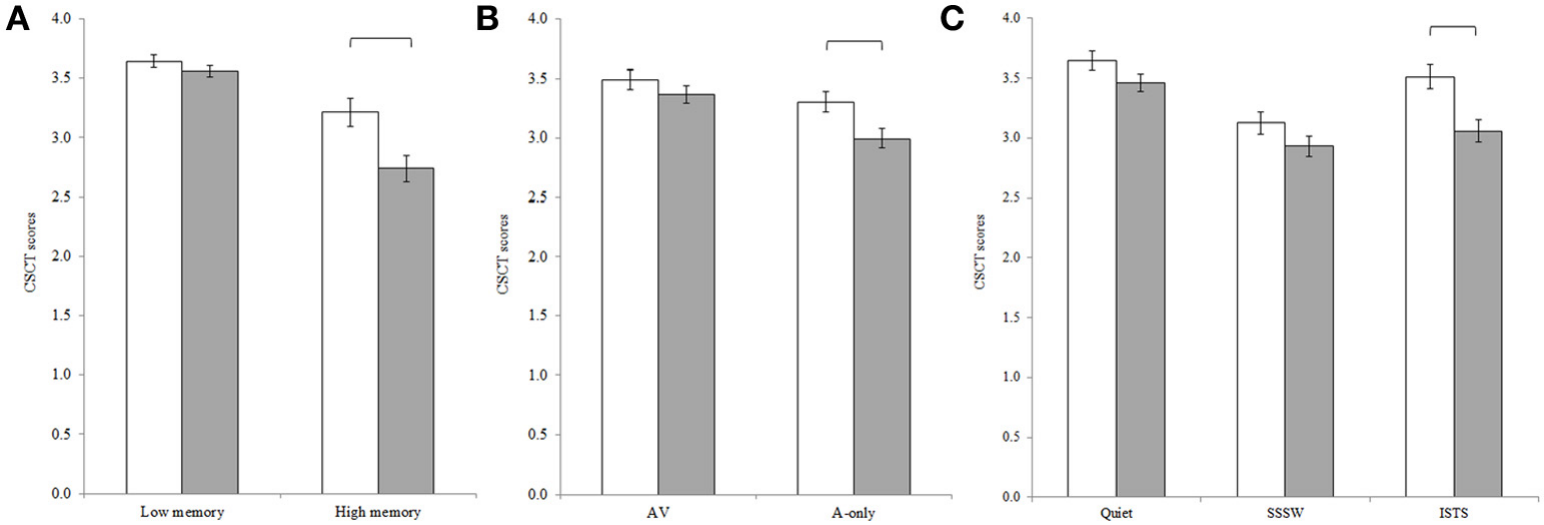
**Two-Way interactions between (A) group and memory load, (B) group and modality and, (C) group and noise, where mean CSCT raw scores for younger adults (unfilled bars) and older adults (filled bars) are shown**. Error bars represent standard error.

The repeated measures ANOVA revealed a main effect of group, *F*_(1, 42)_ = 7.78, *MSE* = 0.02, *p* < 0.01, showing that the participants in the present study had lower CSCT scores compared to young adults with normal hearing thresholds. In line with results for the two groups separately, main effects of executive function, *F*_(1, 42)_ = 35.80, *MSE* = 0.25, *p* < 0.001; memory load, *F*_(1, 42)_ = 93.44, *MSE* = 0.29, *p* < 0.001; modality, *F*_(1, 42)_ = 26.31, *MSE* = 0.12, *p* < 0.001, and noise, *F*_(2, 84)_ = 37.84, *MSE* = 0.18, *p* < 0.001, were observed. Pair-wise comparisons with Bonferroni adjustment for multiple comparisons revealed that the CSCT scores in quiet were significantly higher than the scores in ISTS noise (*p* = 0.001) which in turn were significantly higher than the scores in SSSW noise (*p* < 0.001). The Two-Way interactions between group and memory load [*F*_(1, 42)_ = 7.52, *MSE* = 0.29, *p* < 0.01], group and modality [*F*_(1, 42)_ = 5.07, *MSE* = 0.12, *p* < 0.05], and group and noise [*F*_(2, 84)_ = 3.64, *MSE* = 0.19, *p* < 0.05] were statistically significant, see Figure 3. *Post-hoc* Tukey HSD tests assessing these Two-Way interactions revealed, in line with our predictions, that although the participants in the present study had significantly lower CSCT scores compared to the younger adults in high memory load conditions, there was no statistically significant difference in performance between groups in low load conditions. Further, although participants in the present study had significantly lower CSCT scores in the A-only modality than the younger adults, there was no statistically significant difference in performance between groups in AV conditions. Participants in the present study had lower CSCT scores than younger adults in ISTS noise but there was no statistically significant difference in performance between groups in quiet or in SSSW noise.

**Figure 4:**
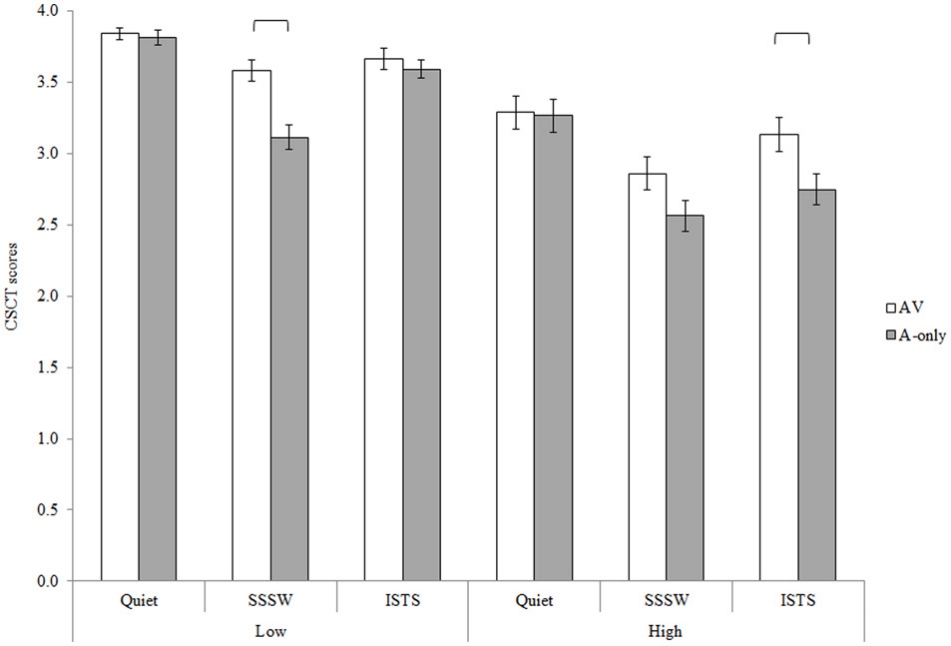
**Three-Way interaction between memory load, modality, and noise for CSCT scores collapsed across groups of participants and the executive task performed**. Error bars represent standard error.

The Two-Way interaction between modality and noise [*F*_(2, 84)_ = 9.25, *MSE* = 0.11, *p* < 0.01] and the Three-Way interaction between memory load, modality and noise [*F*_(2, 84)_ = 3.48, *MSE* = 0.13, *p* < 0.05], see Figure 4, were also statistically significant. Neither of these interactions interacted further with group.

*Post-hoc* Tukey's HSD tests investigating the Two-Way interaction between modality and noise revealed that visual cues significantly enhanced performance in SSSW noise (*p* < 0.01) and ISTS noise (*p* < 0.01) but not in quiet. Investigation of the Three-Way interaction revealed that the findings of the Two-Way interaction were modulated by memory load. In particular, although visual cues enhanced CSCT scores in ISTS noise (*p* < 0.05) when memory load was high, this was not the case when memory load was low. Visual cues enhanced CSCT scores in SSSW noise (*p* < 0.01) in low memory load conditions, but in high memory load conditions the difference in scores did not reach significance with *post-hoc* testing.

#### Cognitive test battery

Independent sample *t*-tests showed that, in line with our prediction, the performance of the participants in the present study was significantly poorer in all the cognitive tests than that of the young adults with normal hearing thresholds (Mishra et al., 2013b, see Table 1). We also found that the mean reaction time for congruent trials in the Simon task in the present study was significantly longer than that found for the younger adults statistically, showing that the participants in the present study had a slower cognitive processing speed than the young adults.

### Discussion

On combining the data of CSCT performance by the older adults with hearing loss in the present study and the young adults without hearing loss in the previous study (Mishra et al., 2013b), we found a main effect of group revealing lower CSCT scores for the older adults with hearing loss compared to the younger adults without hearing loss. Even though intelligibility was held relatively constant, it is likely that the background noise placed a greater burden on cognitive resources in the older adults with hearing loss because of low level auditory processing deficits (Festen and Plomp, 1990; George et al., 2006, 2007; Ben-David et al., 2012). As expected, the older group also performed worse on the cognitive test battery (c.f. Salthouse, 1980; Rönnberg, 1990; Pichora-Fuller and Singh, 2006). Thus, lower CSC for older adults with hearing loss is probably due both to poorer fundamental cognitive abilities and more pressure on those abilities while listening in noise. Examination of the Two-Way interactions with the group factor revealed that the poorer performance of the older adults was driven mainly by performance differences in more challenging conditions: high memory load, A-only modality of presentation and in ISTS noise, in line with our prediction. Across groups, the benefit of visual cues was evident only in noise. It is likely that the visual cues make it easier to distinguish target speech from background noise thus reducing the demand for executive resources during segregation (Besle et al., 2004; Helfer and Freyman, 2005; Mishra et al., 2013b; Moradi et al., 2013). When executive resources are spared during listening, more are likely to be available for solving the CSCT, leading to better performance. In other words, visual cues enhance CSC during listening in noise by freeing executive resources. However, the benefit of visual cues in noise was modulated by memory load and thus further work is needed to investigate the interplay of visual cues, memory load and noise.

## General discussion

The findings of the present study further our understanding of CSC. Using the CSCT, we found lower CSC in older adults with hearing loss compared to the younger adults with normal hearing whom we had tested with the same experimental paradigm in a previous study (Mishra et al., 2013b). This was despite the fact that amplification was provided to compensate for hearing loss and that SNR was individualized to ensure intelligibility. Performance was also significantly poorer on all of the tests in the cognitive test battery. Thus, we suggest that poorer CSC in older adults with hearing loss compared to younger adults with normal hearing is probably due both to poorer cognitive abilities and more pressure on those abilities, rather than differences in hearing thresholds.

As we had predicted, factors previously found to decrease CSC in young adults with normal hearing (Mishra et al., 2013b) had an even stronger effect on the older individuals with hearing loss. In particular, increasing memory load and removing visual cues reduced CSC more for the older group. Further, whereas, steady state noise, but not speech-like, noise reduced CSC for the younger participants in our previous study (Mishra et al., 2013b), both kinds of noise reduced CSC for the older participants in the present study. Lower CSC for older adults with hearing loss in speech-like noise is likely to be related to the poorer ability to segregate target speech from non-target speech, that is well-attested in the literature. This poorer ability probably has several causes at a number of levels including less efficient processing of target information present in the gaps in the speech-like masker (George et al., 2007) poorer inhibition of irrelevant speech (Ben-David et al., 2012), impoverished encoding of target stimuli (Pichora-Fuller et al., 1995; Heinrich and Schneider, 2011; Sörqvist and Rönnberg, 2012) lower WMC (Nyberg et al., 2012) and differences in deployment of cognitive resources during speech understanding (Pichora-Fuller et al., 1995; Murphy et al., 2000; Wong et al., 2009). Further research is needed to tease apart these effects.

The finding of a stronger effect of increasing memory load on CSC for older adults with hearing loss compared to younger adults with normal hearing is in tune with work showing differences in the deployment of cognitive resources for younger and older adults in response to changes in memory load. In particular, imaging studies have shown that older adults display a greater increase in brain activity in response to cognitive load than younger adults, and that brain activity reaches a plateau at a lower level in older adults than in younger adults (Grady, 2012).

Importantly, visual cues enhanced CSC for older adults with hearing loss. Further, this effect was stronger for this group than for young adults with normal hearing. This finding demonstrates that for older adults with hearing loss, visual cues can support the kind of executive processing of speech that may be used in everyday conversation (c.f. Frtusova et al., 2013), possibly because of viseme and phoneme information working together (Feld and Sommers, 2009). Because intelligibility was held relatively constant between groups while cognitive skills were poorer for the older group, it is likely that the mechanism behind this phenomenon is related to cognitive processes.

It is worth noticing that although the older adults with hearing loss generally had reduced CSC compared to young adults; this did not apply in all conditions. When CSCT tasks were performed in quiet, with low memory load and in presence of visual cues, there was no significant difference across groups. This suggests that when listening conditions are optimized, there is no difference in CSC between older adults with hearing loss and younger adults with normal hearing.

In the present study, we found that better CSCT performance in older adults with hearing loss was associated with better updating skills, but only in those conditions in which the participants performed worse than the younger adults with normal hearing who took part in our previous study using the same experimental paradigm (Mishra et al., 2013b). In that study, we found that updating skills predicted CSCT performance in virtually all conditions and suggested that updating skills became particularly important in CSC when inhibition resources are depleted by constantly being prepared to cope with background noise. In the present study, we found that better episodic LTM was associated with better CSCT performance. This pattern of findings further supports the notion that younger adults with normal hearing and older adults with hearing loss deploy cognitive resources differently, especially in relation to changes in task difficulty (Pichora-Fuller et al., 1995; Murphy et al., 2000; Wong et al., 2009; Grady, 2012). In particular, it suggests that LTM may form a processing bottleneck for this group (c.f. Lin et al., 2011) and that more efficient LTM may allow more efficient executive processing of speech with less depletion of CSC (Rönnberg et al., 2013).

## Conclusion

Older adults with hearing loss have lower CSC than young adults without hearing loss, probably because they have poorer cognitive skills and deploy them differently. However, visual cues and efficient episodic LTM enhance CSC more for the older group.

### Conflict of interest statement

The authors declare that the research was conducted in the absence of any commercial or financial relationships that could be construed as a potential conflict of interest.

## References

[B1] Akeroyd M. A. (2008). Are individual differences in speech perception related to individual differences in cognitive ability? A survey of twenty experimental studies with normal and hearing impaired adults. Int. J. Audiol. 47, S53–S71 10.1080/14992020802301142 19012113

[B2] Arehart K. H. Souza P. Baca R. Kates J. M. (2013). Working memory, age, and hearing loss: susceptibility to hearing aid distortion. Ear Hear. 34, 251–260 10.1097/AUD.0b013e318271aa5e 23291963 PMC3636195

[B3] Avivi-Reich M. Daneman M. Schneider B. A. (2014). How age and linguistic competence alter the interplay of perceptual and cognitive factors when listening to conversations in a noisy environment. Front. Syst. Neurosci. 8:21 10.3389/fnsys.2014.00021 24578684 PMC3933794

[B4] Baddeley A. (2003). Working memory: looking back and looking forward. Nat. Rev. Neurosci. 4, 829–839 10.1038/nrn1201 14523382

[B5a] Baddeley A. Hitch G. (1974). Working memory, in Recent Advances in Learning and Motivation, Vol. 8, ed Bower G. A. (New York, NY: Academic Press), 47–90

[B6] Ben-David B. M. Tse V. Y. Schneider B. A. (2012). Does it take older adults longer than younger adults to perceptually segregate a speech target from a background masker? Hear. Res. 290, 55–63 10.1016/j.heares.2012.04.022 22609772

[B7] Besle J. Fort A. Delpuech C. Giard M. H. (2004). Bimodal speech: early suppressive visual effects in the human auditory cortex. Eur. J. Neurosci. 20, 2225–2234 10.1111/j.1460-9568.2004.03670.x 15450102 PMC1885424

[B8] Besser J. Koelewijn T. Zekveld A. A. Kramer S. E. Festen J. M. (2013). How linguistic closure and verbal working memory relate to speech recognition in noise—a review. Trends Amplif. 17, 75–93 10.1177/1084713813495459 23945955 PMC4070613

[B9] Campbell R. (2009). The processing of audiovisual speech: empirical and neural bases, in The Perception of Speech: From Sound to Meaning, 1st Edn., eds Moore B. C. J. Tyler L. K. Marslen-Wilson W. (Oxford, NY: Oxford University Press), 133–150

[B10] Classon E. Rudner M. Rönnberg J. (2013). Working memory compensates for hearing related phonological processing deficit. J. Commun. Disord. 46, 17–29 10.1016/j.jcomdis.2012.10.001 23157731

[B11] Craik F. I. Rose N. S. (2012). Memory encoding and aging: a neurocognitive perspective. Neurosci. Biobehav. Rev. 36, 1729–1739 10.1016/j.neubiorev.2011.11.007 22155274

[B12] Cruickshanks K. J. Wiley T. L. Tweed T. S. Klein B. E. Klein R. Mares-Perlman J. A. (1998). Prevalence of hearing loss in older adults in Beaver Dam, Wisconsin. The epidemiology of hearing loss study. Am. J. Epidemiol. 148, 879–886 10.1093/oxfordjournals.aje.a009713 9801018

[B13] Daneman M. Carpenter P. A. (1980). Individual differences in working memory and reading. J. Verbal Learn. Verbal Behav. 19, 450–466 10.1016/S0022-5371(80)90312-6

[B14] Feld J. Sommers M. (2009). Lipreading, processing speed, and working memory in younger and older adults. J. Speech Lang. Hear. Res. 52, 1555–1565 10.1044/1092-4388(2009/08-0137) 19717657 PMC3119632

[B15] Festen J. M. Plomp R. (1990). Effects of fluctuating noise and interfering speech on the speech reception threshold for impaired and normal hearing. J. Acoust. Soc. Am. 88, 1725–1736 10.1121/1.400247 2262629

[B16] Forster K. I. Forster J. C. (2003). DMDX: a windows display program with millisecond accuracy. Behav. Res. Methods Instrum. Comput. 35, 116–124 10.3758/BF03195503 12723786

[B17] Fraser S. Gagné J.-P. Alepins M. Dubois P. (2010). Evaluating the effort expended to understand speech in noise using a dual-task paradigm: the effects of providing visual speech-cues. J. Speech Lang. Hear. Res. 53, 18–33 10.1044/1092-4388(2009/08-0140) 19635945

[B18] Frtusova J. B. Winneke A. H. Phillips N. A. (2013). ERP evidence that auditory-visual speech facilitates working memory in younger and older adults. Psychol. Aging 28, 481–494 10.1037/a0031243 23421321

[B19] George E. L. J. Festen J. M. Houtgast T. (2006). Factors affecting masking release for speech in modulated noise for normal-hearing and hearing impaired listeners. J. Acoust. Soc. Am. 120, 2295–2311 10.1121/1.2266530 17069325

[B20] George E. L. J. Zekveld A. A. Kramer S. E. Goverts S. T. Festen J. M. Houtgast T. (2007). Auditory and nonauditory factors affecting speech reception in noise by older listeners. J. Acoust. Soc. Am. 121, 2362–2375 10.1121/1.2642072 17471748

[B21] Gordon-Salant S. (2005). Hearing loss and aging: new research findings and clinical implications. J. Rehabil. Res. Dev. 42, 9–24 10.1682/JRRD.2005.01.0006 16470462

[B22] Gosselin P. A. Gagné J.-P. (2011). Older adults expend more listening effort than young adults recognizing audiovisual speech in noise. Int. J. Audiol. 50, 786–792 10.3109/14992027.2011.599870 21916790

[B23] Grady C. (2012). The cognitive neuroscience of ageing. Nat. Rev. Neurosci. 13, 491–505 10.1038/nrn3256 22714020 PMC3800175

[B24] Grimm G. Herzke T. Berg D. Hohmann V. (2006). The master hearing aid: a PC-based platform for algorithm development and evaluation. Acta Acust. United Ac. 92, 618–628

[B25] Hällgren M. Larsby B. Arlinger S. (2006). A Swedish version of the Hearing in Noise Test (HINT) for measurement of speech recognition. Int. J. Audiol. 45, 227–237 10.1080/14992020500429583 16684704

[B26] Heinrich A. Schneider B. A. (2011). The effect of presentation level on memory performance. Ear Hear. 32, 524–532 10.1097/AUD.0b013e31820a0281 21278574

[B27] Helfer K. S. Freyman R. L. (2005). The role of visual speech cues in reducing energetic and informational masking. J. Acoust. Soc. Am. 117, 842–849 10.1121/1.1836832 15759704

[B28] Holube I. Fredelake S. Vlaming M. Kollmeier B. (2010). Development and analysis of an international speech test signal (ISTS). Int. J. Audiol. 49, 891–903 10.3109/14992027.2010.506889 21070124

[B29] Humes L. E. (2007). The contributions of audibility and cognitive factors to the benefit provided by amplified speech to older adults. J. Am. Acad. Audiol. 18, 590–603 10.3766/jaaa.18.7.6 18236646

[B30] Johansson M. S. K. Arlinger S. (2003). Prevalence of hearing impairment in population in Sweden. Int. J. Audiol. 42, 18–28 10.3109/14992020309056081 12564512

[B31] Just M. A. Carpenter P. A. (1992). A capacity theory of comprehension: individual differences in working memory. Psychol. Rev. 99, 122–149 10.1037/0033-295X.99.1.122 1546114

[B32] Lavie N. (2005). Distracted and confused? Selective attention under load. Trends Cogn. Sci. 9, 75–82 10.1016/j.tics.2004.12.004 15668100

[B33] Levitt H. (1971). Transformed up-down methods in psychoacoustics. J. Acoust. Soc. Am. 49, 467–477 5541744

[B34] Lin F. R. Ferrucci L. Metter E. J. An Y. Zonderman A. B. Resnick S. M. (2011). Hearing loss and cognition in the baltimore longitudinal study of aging. Neuropsychology 25, 763 10.1037/a0024238 21728425 PMC3193888

[B35] Lorenzi C. Gilbert G. Carn H. Garnier S. Moore B. C. J. (2006). Speech perception problems of the hearing impaired reflect inability to use temporal fine structure. Proc. Natl. Acad. Sci. U.S.A. 103, 18866–18869 10.1073/pnas.0607364103 17116863 PMC1693753

[B36] Luce P. A. Pisoni D. B. (1998). Recognizing spoken words: the neighborhood activation model. Ear Hear. 19, 1–36 10.1097/00003446-199802000-00001 9504270 PMC3467695

[B37] Lunner T. (2003). Cognitive function in relation to hearing aid use. Int. J. Audiol. 42, S49–S58 10.3109/14992020309074624 12918610

[B38] Mattys S. L. Davis M. H. Bradlow A. R. Scott S. K. (2012). Speech recognition in adverse conditions: a review. Lang. Cogn. Process. 27, 953–978 10.1080/01690965.2012.705006

[B39] McCabe D. P. Roediger H. L. III McDaniel M. A. Balota D. A. Hambrick D. Z. (2010). The relationship between working memory capacity and executive functioning: evidence for a common executive attention construct. Neuropsychology, 24, 222–243 10.1037/a0017619 20230116 PMC2852635

[B40] Mishra S. Lunner T. Stenfelt S. Rönnberg J. Rudner M. (2013a). Visual information can hinder working memory processing of speech. J. Speech Lang. Hear. Res. 56, 1120–1132 10.1044/1092-4388(2012/12-0033) 23785180

[B41] Mishra S. Lunner T. Stenfelt S. Rönnberg J. Rudner M. (2013b). Seeing the talker's face supports executive processing of speech in steady state noise. Front. Syst. Neurosci. 7:96 10.3389/fnsys.2013.00096 24324411 PMC3840300

[B42] Miyake A. Friedman N. P. Emerson M. J. Witziki A. H. Howerter A. Wager T. (2000). The unity and diversity of executive functions and their contribution to complex frontal lobe tasks: a latent variable analysis. Cogn. Psychol. 41, 49–100 10.1006/cogp.1999.0734 10945922

[B43] Moore B. C. Glasberg B. R. (1998). Use of a loudness model for hearing-aid fitting. I. Linear hearing aids. Br. J. Audiol. 32, 317–335 10.3109/03005364000000083 9845030

[B44] Moradi S. Lidestam B. Rönnberg R. (2013). Gated audiovisual speech identification in silence versus noise: effect on time and accuracy. Front. Psychol. 4:359 10.3389/fpsyg.2013.00359 23801980 PMC3685792

[B45] Morris N. Jones D. M. (1990). Memory updating in working memory: the role of the central executive. Br. J. Psychol. 81, 111–121 10.1111/j.2044-8295.1990.tb02349.x

[B46] Murphy D. R. Craik F. I. M. Li K. Z. H. Schneider B. A. (2000). Comparing the effects of aging and background noise on short-term memory performance. Psychol. Aging 15, 323–334 10.1037/0882-7974.15.2.323 10879586

[B48] Nilsson L. G. Backman L. Erngrund K. Nyberg L. Adolfsson R. Bucht G. (1997). The Betula prospective cohort study: memory, health, and aging. Aging Neuropsychol. Cogn. 4, 1–32 10.1080/13825589708256633

[B49] Nyberg L. Lövdén M. Riklund K. Lindenberger U. Bäckman L. (2012). Memory aging and brain maintenance. Trends Cogn. Sci. 16, 292–305 10.1016/j.tics.2012.04.005 22542563

[B50] Pichora-Fuller M. K. (2003). Processing speed and timing in aging adults: psychoacoustics, speech perception, and comprehension. Int. J. Audiol. 42, S59–S67 10.3109/14992020309074625 12918611

[B51] Pichora-Fuller M. K. (2007). Audition and cognition: what audiologists need to know about listening, in Hearing Care for Adults, eds Palmer C. Seewald R. (Stäfa: Phonak), 71–85

[B52] Pichora-Fuller M. K. Schneider B. A. Daneman M. (1995). How young and old adults listen to and remember speech in noise. J. Acoust. Soc. Am. 97, 593–608 10.1121/1.412282 7860836

[B53] Pichora-Fuller M. K. Singh G. (2006). Effects of age on auditory and cognitive processing: implications for hearing aid fitting and audiologic rehabilitation. Trends Amplif. 10, 29–59 10.1177/108471380601000103 16528429 PMC4111543

[B54] Pichora-Fuller M. K. Souza P. E. (2003).Effects of aging on auditory processing of speech. Int. J. Audiol. 42, S11–S16 10.3109/14992020309074638 12918623

[B55] Pratte M. S. Rouder J. N. Morey R. D. Feng C. (2010). Exploring the differences in distributional properties between Stroop and Simon effects using delta plots. Atten. Percept. Psychophys. 72, 2013–2025 10.3758/APP.72.7.2013 20952797

[B56] Reuter-Lorenz P. A. Cappell K. A. (2008). Neurocognitive aging and the compensation hypothesis. Curr. Dir. Psychol. Sci. 17, 177–182 10.1111/j.1467-8721.2008.00570.x

[B57] Rönnberg J. (1990). Cognitive and communication function: the effects of chronological age and “handicap age.” Eur. J. Cogn. Psychol. 2, 253–273 10.1080/09541449008406207

[B58] Rönnberg J. (2003). Cognition in the hearing impaired and deaf as a bridge between signal and dialogue: a framework and a model. Int. J. Audiol. 42, S68–S76 10.3109/14992020309074626 12918612

[B59] Rönnberg J. Arlinger S. Lyxell B. Kinnefors C. (1989). Visual evoked potentials: relation to adult speechreading and cognitive function. J. Speech Lang. Hear. Res. 32, 725–735 2601304

[B60] Rönnberg J. Danielsson H. Rudner M. Arlinger S. Sternäng O. Wahlin A. (2011). Hearing loss is negatively related to episodic and semantic long-term memory but not to short-term memory. J. Speech Lang. Hear. Res. 54, 705–726 10.1044/1092-4388(2010/09-0088) 20884779

[B61] Rönnberg J. Lunner T. Zekveld A. Sörqvist P. Danielsson H. Lyxell B. (2013). The ease of language understanding (ELU) model: theoretical, empirical and clinical advances. Front. Syst. Neurosci. 7:31 10.3389/fnsys.2013.00031 23874273 PMC3710434

[B62] Rönnberg J. Rudner M. Foo C. Lunner T. (2008). Cognition counts: a working memory system for ease of language understanding (ELU), Int. J. Audiol. 47, S99–S105 10.1080/14992020802301167 19012117

[B63] Rudner M. Foo C. Rönnberg J. Lunner T. (2011). Working memory supports listening in noise for persons with hearing impairment. J. Am. Acad. Audiol. 22, 156–167 10.3766/jaaa.22.3.4 21545768

[B64] Rudner M. Lunner T. (2013). Cognitive spare capacity as a window on hearing aid benefit. Semin. Hear. 34, 297–306 10.1055/s-0033-1356642

[B65] Salthouse T. (1980). Age and memory: strategies for localizing the loss, in New Direction in Memory and Aging, eds Poon L. W. Fozard J. L. Cermak L.S. Arenberg D. Thompson L.W. (Hillsdale, NJ: Lawrence Erlbaum Associates Inc), 47–65

[B66] Simon J. R. (1969). Reactions towards the source of stimulation. J. Exp. Psychol. 81, 174–176 10.1037/h0027448 5812172

[B67] Sörqvist P. Ljungberg J. K. Ljung R. (2010). A sub-process view of working memory capacity: evidence from effects of speech on prose memory. Memory 18, 310–326 10.1080/09658211003601530 20182946

[B68] Sörqvist P. Rönnberg J. (2012). Episodic long-term memory of spoken discourse masked by speech: what role for working memory capacity? J. Speech Lang. Hear. Res. 55, 210–218 10.1044/1092-4388(2011/10-0353) 22199182

[B69] Studebaker G. A. (1985). A “rationalized” arcsine transform. J. Speech Lang. Hear. Res. 28, 455–462 4046587 10.1044/jshr.2803.455

[B70] Weatherly S. L. (2002). Back to basics: testing visual acuity with the Jaeger eye chart. Mater. Eval. 60, 928–929

[B71] WHO. (2013). Available online at: http://www.who.int/pbd/deafness/hearing_impairment_grades/en/

[B72] Wong P. C. Jin J. X. Gunasekera G. M. Abel R. Lee E. R. Dhar S. (2009). Aging and cortical mechanisms of speech perception in noise. Neuropsychologia. 47, 693–703 10.1016/j.neuropsychologia.2008.11.032 19124032 PMC2649004

[B73] Zekveld A. A. George E. L. J. Kramer S. E. Goverts S. T. Houtgast T. (2007). The development of the text reception threshold test: a visual analogue of the Speech reception threshold test. J. Speech Lang. Hear. Res. 50, 576–584 10.1044/1092-4388(2007/040) 17538101

[B74] Zekveld A. A. Rudner M. Johnsrude I. S. Heslenfeld D. J. Rönnberg J. (2012). Behavioral and fMRI evidence that cognitive ability modulates the effect of semantic context on speech intelligibility. Brain Lang. 122, 103–113 10.1016/j.bandl.2012.05.006 22728131

[B75] Zekveld A. A. Rudner M. Johnsrude I. S. Rönnberg J. (2013). The effects of working memory capacity and semantic cues on the intelligibility of speech in noise. J. Acoust. Soc. Am. 134, 2225–2234 10.1121/1.4817926 23967952

[B76] Zion Golumbic E. M. Ding N. Bickel S. Lakatos S. Schevon C. A. McKhann G. M. (2013). Mechanism underlying selective neuronal tracking of attended speech at a “cocktail party.” Neuron 77, 980–991 10.1016/j.neuron.2012.12.037 23473326 PMC3891478

